# Accuracy of implant surgery with surgical guide by inexperienced clinicians: an in vitro study

**DOI:** 10.1002/cre2.3

**Published:** 2015-07-14

**Authors:** Takeshi Toyoshima, Hideaki Tanaka, Masanori Sasaki, Eiji Ichimaru, Yasushi Naito, Yasuyuki Matsushita, Kiyoshi Koyano, Seiji Nakamura

**Affiliations:** ^1^ Division of Maxillofacial Diagnostic and Surgical Sciences, Faculty of Dental Science Kyushu University Fukuoka Japan; ^2^ Regenerative Dentistry and Implant Center Kyushu University Hospital Fukuoka Japan; ^3^ Ichimaru Dental Office Kuranoue Shin‐Tosu Periodontal and Implant Dentistry Center Tosu Japan; ^4^ Division of Oral Rehabilitation, Faculty of Dental Science Kyushu University Fukuoka Japan

**Keywords:** artificial mandible, inexperienced clinician, in vitro, implant surgery, model, surgical guide, unilateral free‐end edentulism

## Abstract

Implant surgery with surgical guide has been introduced with a concept of position improvement. The surgery might be considered as easy even for inexperienced clinician because of step simplicity. However, there are residual risks, resulting in postoperative complications. The aim of this study was to assess the accuracy of implant surgery with surgical guide by inexperienced clinicians in in vitro. After preoperative computed tomographies (CTs) of five artificial models of unilateral free‐end edentulism with scan templates, five surgical guides were established from templates. Following virtual planning, 10 implants were placed in the 45 and 47 regions by five residents without any placement experiences. All drillings and placements were performed using surgical guides. After postoperative CTs, inaccurate verifications between virtual and actual positions of implants were carried out, by overlaying of pre/postoperative CT data. The angle displacement of implant axis in the 47 region was significantly larger than that in the 45 region (*P* = 0.031). The 3D offset of implant base in the 47 region was significantly larger than that in the 45 region (*P* = 0.002). For distal/apical directions, displacements of base in the 47 region were significantly larger than those in the 45 region (*P* = 0.004 and *P* = 0.003, respectively). The 3D offset of implant tip in the 47 region was significantly larger than that in the 45 region (*P* = 0.003). For distal/apical directions, displacements of tip in the 47 region were significantly larger than those in the 45 region (*P* = 0.002 and *P* = 0.003, respectively). Within limitations of this in vitro study, data for accuracy of implant surgery with surgical guide would be informative for further studies, because in vitro studies should be substantially made to avoid unnecessary burden of patients, in advance of retro/prospective studies. A comparison of the accuracy in this in vitro model between by inexperienced and well‐experienced operators should be necessary for clinicians intending to use surgical guide for placement.

## Introduction

Implant surgery with surgical guide gains more and more importance in implant dentistry (Ewers et al. 2005; Hammerle et al. 2009). It is a key goal of the surgery with the guide to obtain a maximum accuracy by transferring the virtual implant position into the clinical situation (Arisan et al. 2010). In reality, the surgery requires several steps as follows; fabrication of a radiographic template, computed tomography (CT) acquisition with the template in position, computer‐assisted planning of implant placement and ending in fabrication, and the use of surgical guide for drilling and implant placement. Thus, the accuracy depends on all cumulative and interactive errors involved from fabrication of a radiographic template to the placement with surgical guide (Miller and Bier 2006; Jung et al. 2009).

Previous studies have proved a high accuracy for implant surgery with surgical guide (Wittwer et al. 2007; Ruppin et al. 2008; Schneider et al. 2009; Vasak et al. 2011). The mean deviation at the apex of 1.0 mm was found in eight patients with partial edentulism by flapless placement with stereolithographic guide (Van Assche et al. 2010). The deviation at the apex was 0.59 mm in the maxilla and 0.4 mm in the mandible in an experimental model with computer‐aided design and computer‐aided manufacturing template (Komiyama et al. 2011). The deviation was 1.09 mm at the coronal and 1.56 mm at the apical in 48 patients with 102 implants by stereoradiographic guide (Lee et al. 2013). The deviation was 0.72 mm at the base and 0.46 mm at the tip in five human cadaver by half‐guided system (Kuhl et al. 2013). The difference between planned and actual entry points to the maxilla ranged between 0.7 and 6.0 mm. Also, in a review of accuracy in computer‐guided surgery (Widmann and Bale 2006), a maximum differences between the planning versus the actual position were found from 1.2 to 2.0 mm. However, at present, there are still no accepted standards for the accuracy of implant surgery with surgical guide. In addition, as the accuracy measurements of the actual position of placed implant versus implant position from vertical planning in vivo involves the postoperative CT acquisition, this is medically unjustifiable in most clinical cases.

Because the implant surgery with surgical guide might be considered to be easy because of the systematic simplicity of surgical steps and the high accuracy previously reported, even less‐experienced clinician would perform this surgery. However, it is also veracious that there is a pitfall with the residual risk of uncontrolled and blind placement of implant, resulting in complications such as perforation to sinus membrane in maxilla or nerve injury in mandible, because this surgery would be usually adapted in order to place correctly implant in severe cases with sensitive structures of anatomy such as maxillary sinus, mandibular canal, and mental foramen. Thus, the exact accuracy of implant surgery using surgical guide by inexperienced clinicians should be verified in in vitro model.

All systems of surgical guide use drill and sleeve combinations for the guidance. Basically, the sleeves guide the drills during preparation of implant cavity, according to the virtual planning (Arisan et al. 2010). In the system employed in this study, there are several advantages for accuracy improvement. First, a fit of surgical guide with tooth or mucosa should be exactly the same with that at the time of preoperative CT acquisition, because a scan template used on the CT acquisition can be customized to a surgical guide for implant surgery. Second, data on the diagnostic software as numerical form can be directly used for customization of surgical guide. Third, a milling cutter can reduce displacement possibility of implant cavity position from virtual planning, because the cutter is initially used to shave bone surface as flat before drilling, and therefore, drills are difficult to slip. Moreover, sleeves can be selected because of interocclusal distance between jaws.

The aim of this study was thus to assess the accuracy of implant surgery with surgical guide by inexperienced clinicians in in vitro.

## Materials and Methods

### Preparation of surgical guide

**Figure 1:**
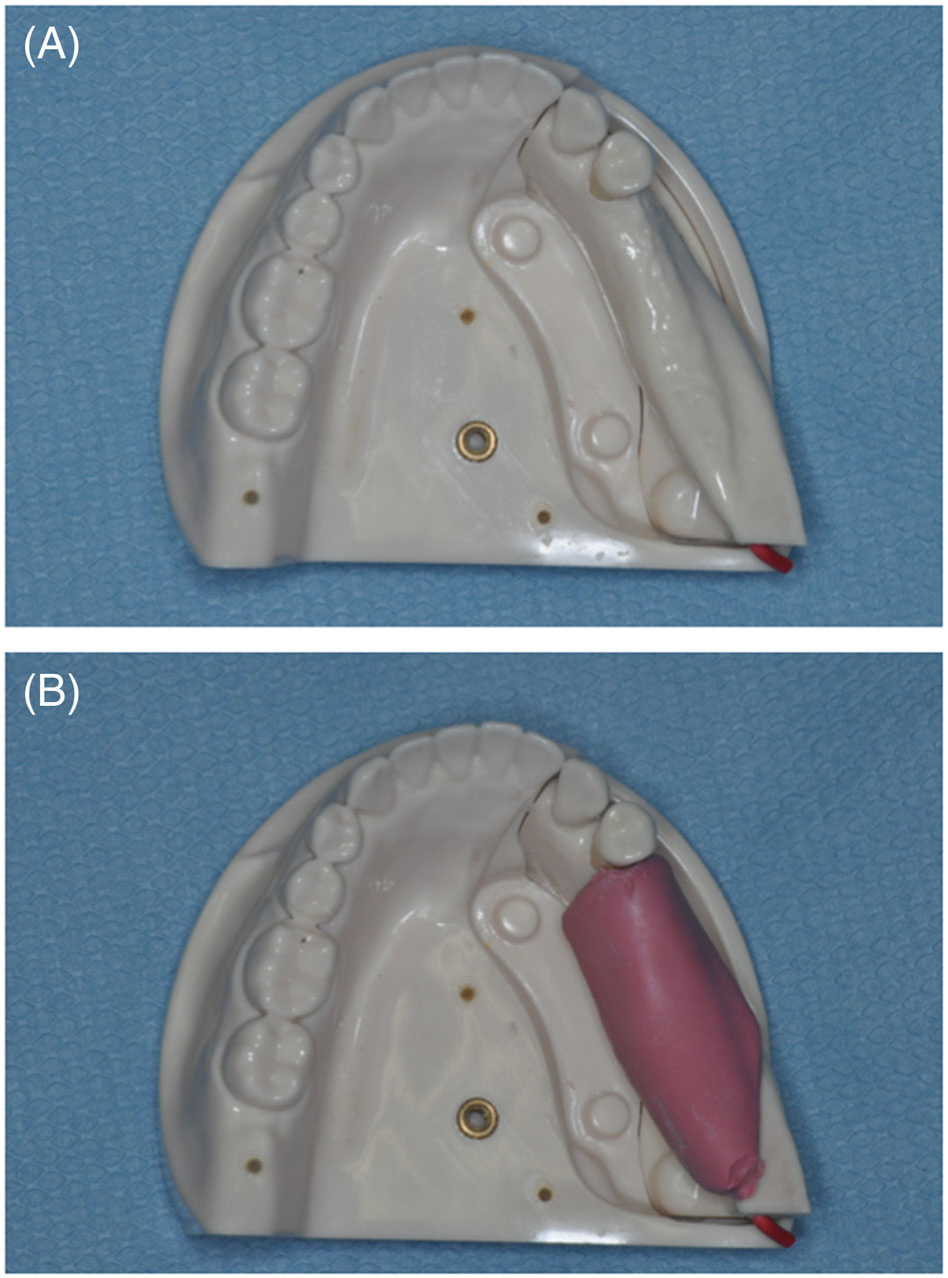
Artificial mandible model of unilateral free‐end edentulism. (A) Occlusal view of the model without artificial mucosa. The part of the 43–47 regions was newly replaced for each experiment based on the rest of one mandible model (37–42 regions). (B) Occlusal view of the model with artificial mucosa. Silicone impression material of a 3‐mm‐thick layer was used as an artificial mucosa on the 45–48 regions.

Five artificial mandible models of unilateral free‐end edentulism (P9‐IMP.6‐L, NISSIN, Kyoto, Japan) were used for this model study. Silicone impression material of a 3‐mm‐thick layer (Examixfine Regular Type, GC, Tokyo, Japan) was also used for artificial mucosa on the 45–48 regions in each model (Fig. 1). Each part of the 43–47 regions was replaced and assembled onto the mother mandibular model (37–42 regions) for each experiment. Five plaster models were fabricated, after taking impressions of each model. Crown forms were mocked‐up the prosthesis planned on the 45–47 regions of unilateral free‐end edentulism and were transformed into five scan templates with 3.0 mm‐thick of Imprelon^®^ by the vacuum former, Ministar S Scan^®^ (Scheu, Iserlohn, Germany). An acrylic plate with three titanium pins located along the inside of dental arch as reference (templiX^TM^; Straumann, Basel, Switzerland) was fixed onto each template. The models with inserted templates were mounted and casted into a GonyX^TM^‐table (Straumann) according to the manufacturer's instructions.

**Figure 2:**
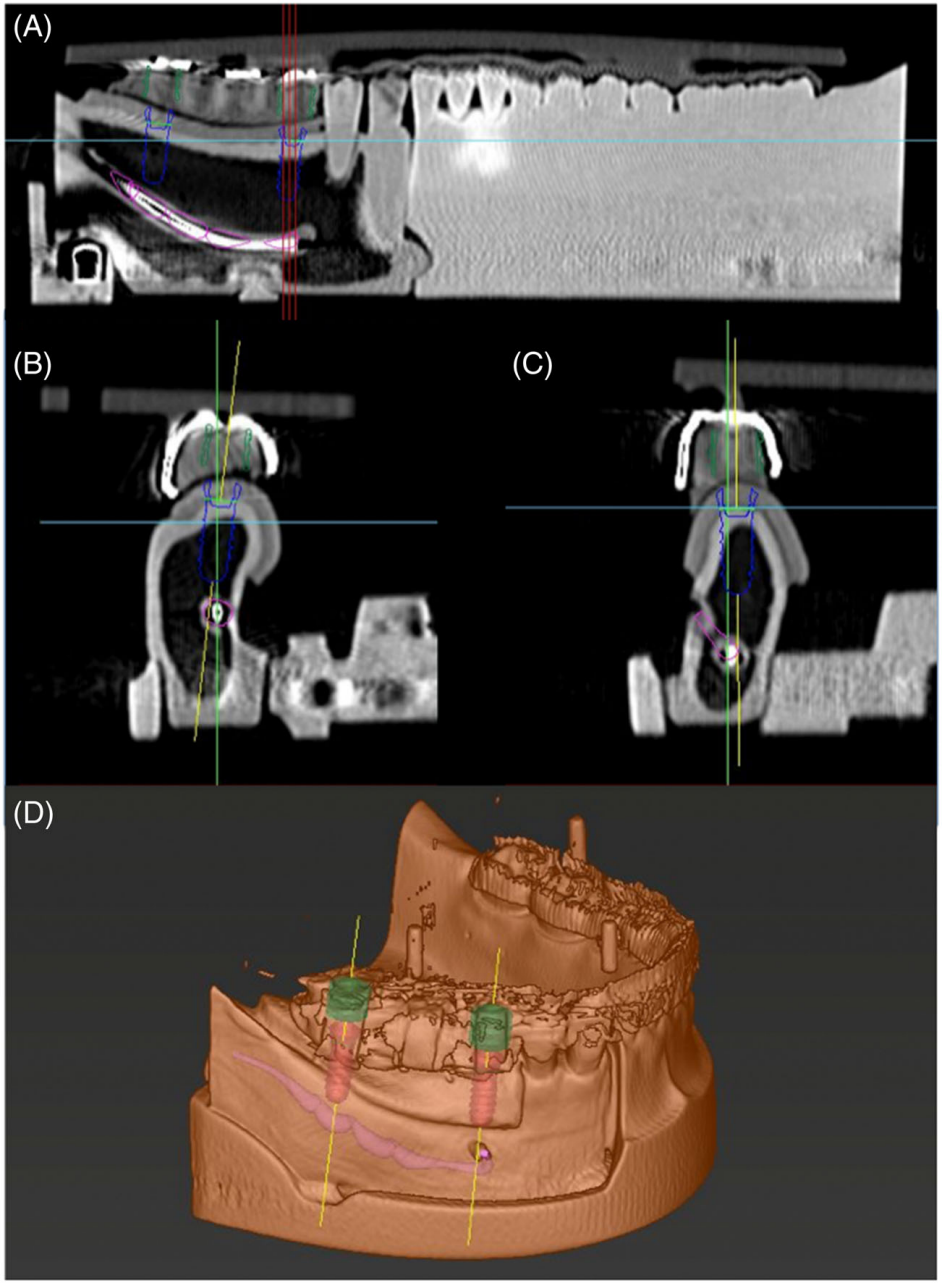
Virtual planning of implant position on the 45 and 47 regions from preoperative CT. (A) Panoramic image on the 45 and 47 regions. (B) Cross‐sectional image on the 47 region. (C) Cross sectional image on the 45 region. (D) 3D image of virtual planning.

**Figure 3:**
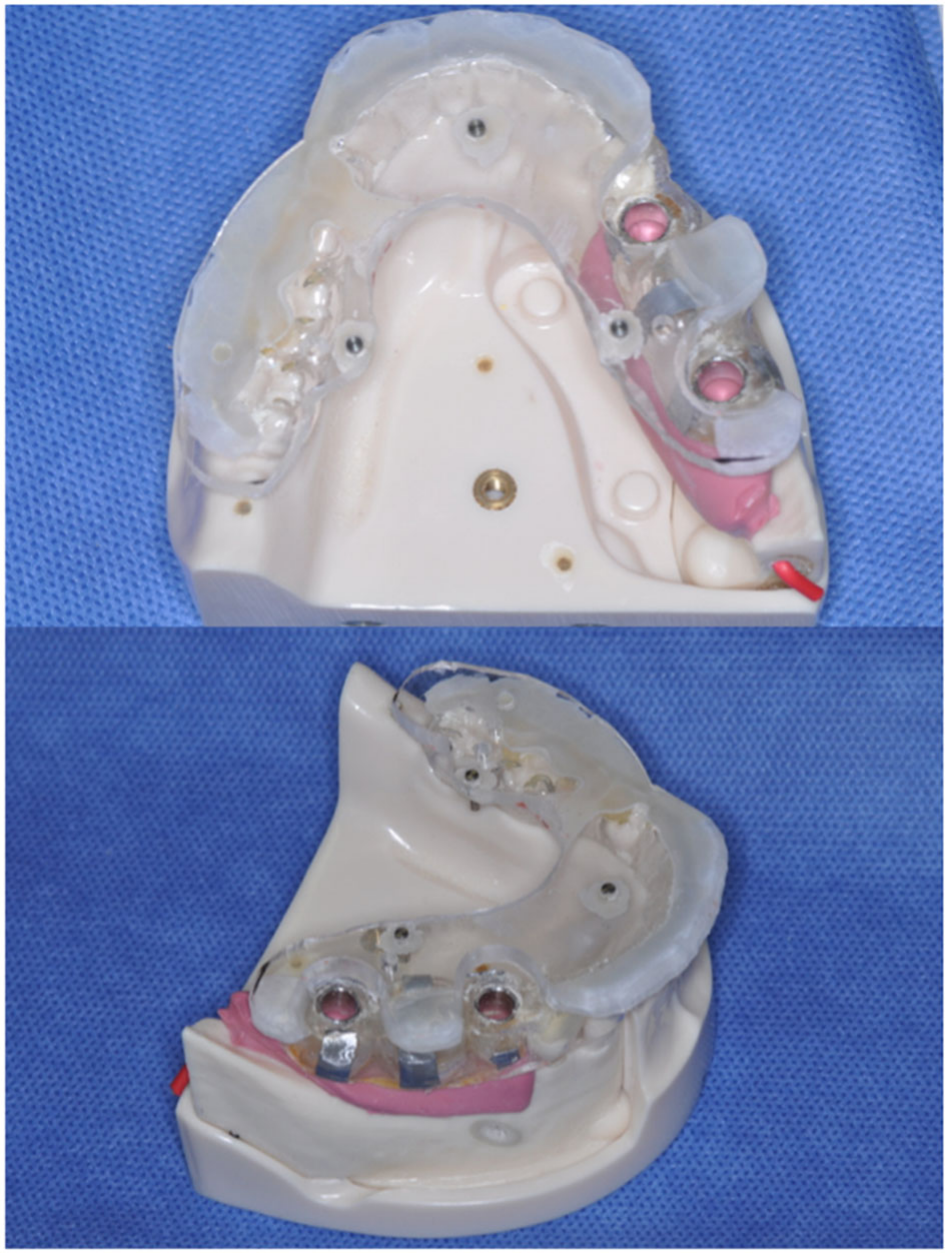
Surgical guide used in this study. An acrylic plate with three titanium pins located along the inside of dental arch as reference (templiX^TM^; Straumann, Basel, Switzerland) was fixed onto each template established by vacuum former. Guiding sleeves of 5‐mm length and 5‐mm diameter were incorporated into the template.

Once casted, the templates were removed from the plaster models, cleaned, and fixed onto the artificial mandible models of unilateral free‐end edentulism. The helical CTs (CB Mercu Ray; Hitachi Medical Corporation,Tokyo, Japan) at standard setting of 120 kV, 15 mA, an exposure time of 9.6 s, and a voxel size of 0.2 mm were preoperatively performed. The correct fit of the templates was visually and manually controlled before the CTs. Templates in the models of unilateral free‐end edentulism were fixed during CTs by friction on the remaining teeth and the mucosa. The dataset of the CTs was transformed into digital imaging and communications in medicine files and uploaded into the planning software (co‐DiagnostiX^TM^, Version 7.03; Straumann). In each dataset, two identical implants (Standard Plus length: 10 mm; diameter 4.1 mm, Straumann) were planned for the 45 and 47 regions virtually on the co‐DiagnostiX^TM^ (Fig. 2). A total of 10 implants were planned. According to the virtual planning and manufacturer's instructions, at the height of 6 mm from the border of machined and rough surface, guiding sleeves of 5 mm length and 5 mm diameter were incorporated into the templates using the co‐DiagnostiX^TM^ and GonyX^TM^ devices, as surgical guides (Fig. 3). The correct position of each sleeve was controlled manually with a printed medial provided by the software system.

### Implant placements

Five residents without any experience of implant placement volunteered to this experiment. As calibration, the definite instruction for placements was given to the residents just before placements to cover the interoperator and intraoperators' variations. The artificial mandible models were set on phantom head model (Nissim Type 1, NISSIN). Each template was positioned into each model, and the precise fit was visually and manually controlled before surgery. The artificial mucosa in the 45–47 regions was removed as incision and flap elevation. Flap elevation followed by implant placements is defined in this guide system. As the surgical guide is exactly lengthened to the distal point of the 47 region from anatomical point of view with the consideration of position of retromolar pad and incision line of mucosa would be posteriorly set over the distal point, the 45–47 regions of surgical guide accordingly lose the mucosal support. Therefore, the mucosa gum was completely removed from the model as incision and flap elevation in this model study. Implant bed preparations were performed according to the manufacturer's instructions using the guided surgery kit (Straumann). The system provides a combination of guided drills with depth control and drill handles based on the tube‐in‐tube concept. Beds of 10 mm depths and 3.5 mm diameter were drilled through the sleeves. Two implants (Guided Standard Plus length: 10 mm; diameter 4.1 mm, Straumann) were placed through the sleeves on the 45–47 regions with guided implants (Guided Standard Plus length: 10 mm; diameter 4.1 mm, Straumann), which controlled the vertical position with a mechanical stop key. Each implant was inserted only through the surgical guide with handpieces without any manual correction.

### Accuracy evaluations

**Figure 4:**
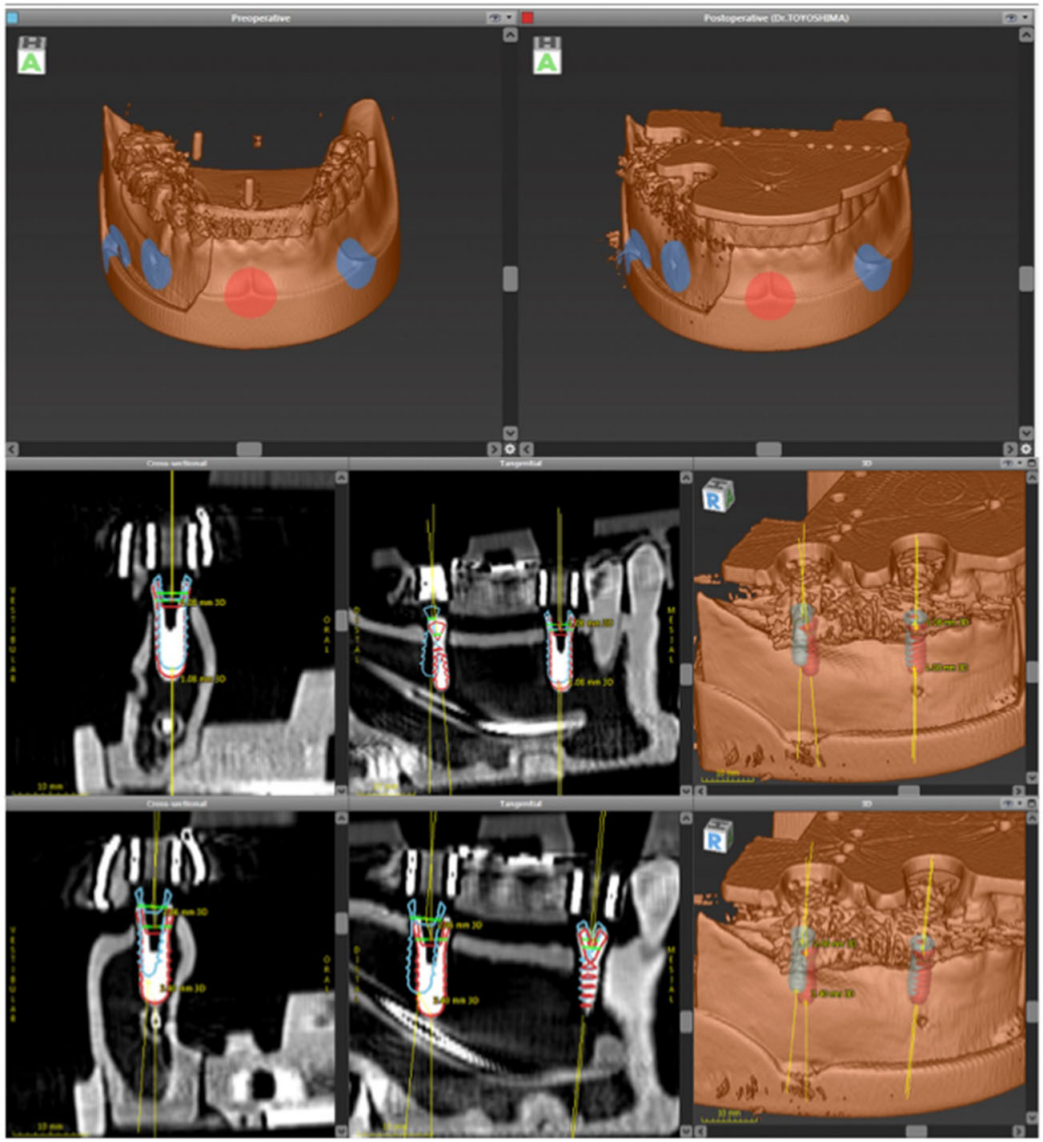
Accuracy evaluations of implants. Postoperative computed tomographies were performed in compliance with the preoperative image acquisition and transformed into the digital imaging and communications in medicine format. The preoperative planning datasets were manually aligned with the postoperative data using rigid registration and anatomical landmarks.

**Figure 5:**
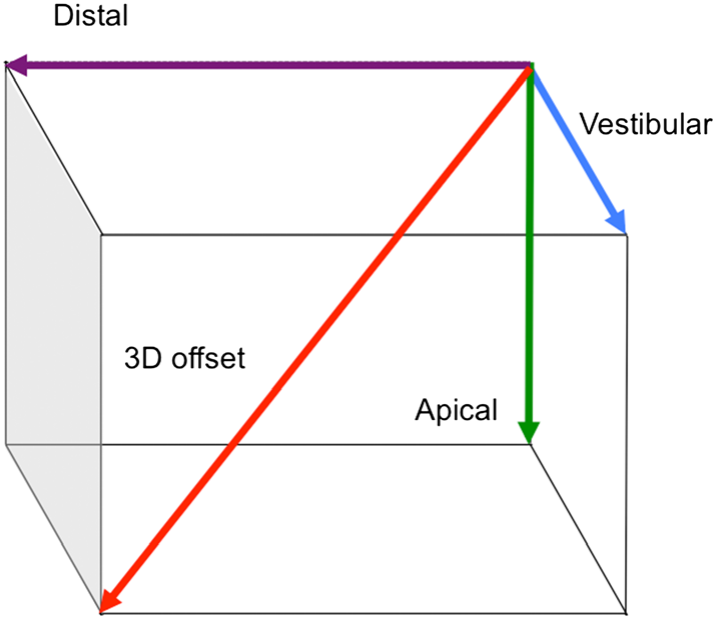
Definition of implant displacement as distal, vestibular, apical direction, and 3D offset for total displacement.

Accuracy evaluations of implants were carried out, according to the manufacturer's instructions. Postoperative CTs were performed in compliance with the preoperative image acquisition and transformed into the digital imaging and communications in medicine format. The preoperative planning datasets were manually aligned with the postoperative data using rigid registration and anatomical landmarks (Fig. 4). To determine the implant tip and base position in the postoperative dataset and the implant axis, the implants were first segmented using region growing. The tip and the base were then located by searching along each implants main axis. The total displacement of the axis was determined comparing the planned and the main axis of the implants. Displacements of implants were defined as distal, vestibular, apical direction, and 3D offset for total displacement (Fig. 5).

### Statistical evaluation

Statistical analyses were performed using spss software (version 17.0, SPSS Inc., Chicago, IL, USA). Statistical significance of the differences between the groups was determined by student *t*‐test. *P*‐values of less than 0.05 were considered to be significant.

## Results

Raw data of displacements of implant bases and tips for 3D offset, angle, distal, vestibular, and apical direction are summarized in Table 1.

**Table 1:** Raw data of displacements of implant bases and tips for 3D offset, angle, distal, vestibular, and apical direction.

Region	Model number	Angle (^°^)	Displacement of base (mm)				Displacement of base (mm)			
			3D offset	Distal	Cestibular	Apical	3D offset	Distal	Cestibular	Apical
45	1	0	1.08	−0.33	0.12	1.03	1.08	−0.33	0.12	1.03
	2	0.9	1.35	0.05	−0.52	1.24	1.42	0.05	−0.68	1.25
	3	6.1	1.20	−0.39	−0.41	1.06	1.98	−1.14	−1.18	1.12
	4	3.2	1.23	0.28	0.04	1.20	1.24	−0.28	0.04	1.21
	5	0.3	2.17	0.01	−0.57	2.09	2.17	−0.04	−0.57	2.09
	Mean ± SD 2.10 ± 2.29		1.41 ± 0.39 − 0.08 ± 0.25 − 0.27 ± 0.29 1.32 ± 0.39				1.58 ± 0.42 − 0.35 ± 0.42 ± 0.45 ± 0.48 1.34 ± 0.38			
47	1	6.5	2.86	−0.71	−0.30	2.76	3.40	−1.65	−0.93	2.82
	2	2.6	2.34	−0.48	−0.26	2.27	2.48	−0.93	−0.26	2.28
	3	6.0	2.50	−0.85	−0.63	2.27	3.14	−1.71	−1.23	2.32
	4	8.5	3.34	−0.44	0.59	3.26	4.01	−1.68	1.38	3.37
	5	3.7	1.99	−0.53	−0.32	1.89	2.20	−1.09	0	1.91
	Mean ± SD 5.46 ± 2.09*		2.61 ± 0.46†−0.60 ± 0.15†−0.18 ± 0.41 2.49 ± 0.47†				3.05 ± 0.65† −1.41 ± 0.33† −0.21 ± −0.91 2.54 ± 0.51†			

* For angle, statistical significance of the differences between mean values of the 47 region and 45 region was determined by the student *t*‐test (*P* < 0.05).

† For 3D offset, distal, and apical direction, statistical significance of the differences between the mean values of the 47 region and 45 region was determined by the student *t*‐test (*P* < 0.01).

The degree of the angle in the 47 region was significantly larger than that in the 45 region (*P* = 0.031). The 3D displacement of base in the 47 region was significantly larger than that in the 45 region (*P* = 0.002). For the distal and the apical directions, displacements of base in the 47 region were significantly larger than those in the 45 region (*P* = 0.004 and *P* = 0.003, respectively), while they were not for the vestibular direction. In a same manner, the 3D displacement of tip in the 47 region was significantly larger than that in the 45 region (*P* = 0.003). For the distal and the apical directions, displacements of tip in the 47 region were significantly larger than those in the 45 region (*P* = 0.002 and *P* = 0.003, respectively), while they were not for the vestibular direction.

## Discussion

To our knowledge, this is the first report to assess the accuracy of implant surgery with surgical guide by inexperienced clinicians in in vitro model. There have not been so many reports about the influence of operator experience on the accuracy of implants with surgical guides. However, it has been frequently demonstrated that operator experience level is related to implant survival rates (Preiskel and Tsolka 1995; Lambert et al. 1997; Sennerby and Roos 1998; Kohavi et al. 2004; Melo et al. 2006; Starr and Maksoud 2006; Van de Velde et al. 2008; Cho et al. 2010; Zoghbi et al. 2011). Lambert et al. found that surgeons who had placed less than 50 implants had almost twice the failure rate as more experienced surgeons. A possible explanation for the poor outcome of implants placed by less‐experienced surgeons is that the frequency of problems such as excessive heat during drilling, non‐stabilization of the implant, lack of adequate planning may increase with less experience. Van de Velde et al. examined the accuracy of implants placed without surgical guides by operators with varying experience as compared to a virtual implant plan (Van de Velde et al. 2008). An experienced operator placed significantly implants in the horizontal and angular dimensions better than an inexperienced operator. Another report has shown that the experience level of the operator contributes to the accuracy of implant placement with surgical guide (Cushen and Turkyilmaz 2013). In this in vitro study, two operators were experienced in implant placements, and the other two had limited experience. A significant difference was found in the accuracy of implants based on the operator experience. Moreover, in a recent in vitro study, the impact of operator experience on the accuracy of implant placement with stereolithographic bone‐supported guides was examined (Cushen and Turkyilmaz 2013). Two operators had measurable experience (over 100 implants placed), and two had little experience (less than 10 implants placed). Statistical analysis showed a significant difference between the experienced and inexperienced group for angular and horizontal error at the implant tip and at the base. The same conclusion could be drawn out in an in vitro study on the accuracy of micro‐implant placement performed by experienced and inexperienced operators (Cho et al. 2010). Furthermore, a recent in vivo study suggests that inexperienced operator had no influence on the accuracy of implant placement with surgical guide in fully edentulous jaws if all steps of the surgery were supervised by experienced clinicians. It means that implant surgery with surgical guide should not be made by inexperienced operator individual. In this study, five residents with no experience of implant placement volunteered to the implant surgeries with surgical guide. In clinical situation, surgical guide would be used for severe cases such as single tooth replacements in esthetic zone, fixed rehabilitations in edentulous maxilla with atrophy, or rehabilitations in mandible with vertical bone deficiency, with aims to make the surgical procedure easy, safer, and more predictable. However, this surgery would be comprehended as easy even for less‐experienced clinician, because of the systematic simplicity of surgical steps and the high accuracy previously reported. Utilization of surgical guides does not justify the inclusion of inexperienced clinicians to place implants in clinical situation, because it cannot compensate for inexperience of clinicians. Thus, initially, the exact accuracy of implant surgery using surgical guide by inexperienced clinicians should be verified in this in vitro model. Although most of the previous reports have compared the accuracy between well‐experienced and less‐experienced operators in vivo retrospective studies, in vitro studies should be substantially made with well‐experienced and inexperienced clinicians in order to avoid any unnecessary burden of patients before retrospective or prospective studies. Therefore, a comparison of the accuracy of implant surgery with surgical guide in this in vitro model between the inexperienced and well‐experienced operators should be necessary for clinicians who would intend to use surgical guide for implant placement. Moreover, a chronological observation of the accuracy could be also productive in further investigations. Clinical reports previously suggest that experience degree of operators would influence on the accuracy of the surgery, but there have been no reports to show chronological changes of operators according to gaining of placement experiences. Therefore, it could be interesting to observe if less‐experienced operator could improve skills to accurately place implants with surgical guide, according to the number increase of implants that they have placed.

The results of this study show that the accuracy of the actual position of placed implant in the 47 region was significantly worse than that in the 45 region in artificial mandible models of unilateral free‐end edentulism. A meta‐analysis showed that the accuracy at the base reveals a mean error of 0.74 mm with a maximum of 4.5 mm and a mean error at the tip of 0.85 mm with a maximum of 7.1 mm (Jung et al. 2009). Another systematic review on the accuracy of guided surgery showed a mean deviation of 1.07 mm at the base and 1.63 mm at the tip (Schneider et al. 2009). Comparing with these results, the accuracy of 3D displacement in the 45 region in this study was slightly worse at the base and the tip with a mean deviation of 1.41 mm and 1.58 mm, respectively. And the accuracy in the 47 region was completely worse at the base and at the tip with a mean deviation of 2.61 and 3.05 mm, respectively. The study using coDiagnostiX^TM^ showed an overall discrepancy of 0.6 mm in the lateral/medial direction of the tip and 0.9 mm in the anterior/posterior direction (Nickenig and Eitner 2010). As these results are better than ours, it should be quite difficult to simply compare one study with another, when model based, cadaver based, and clinical studies were included together. On the other hand, in clinical situation, mobility of teeth supporting surgical guides, mucosal thickness under surgical guides, and mucosal existence by flapless technique should especially influence on accuracy of guides surgery, and it is thus suggested these results obtained in this study cannot be directly traced. These studies came to the conclusion that the accuracy depends on all cumulative and interactive errors involved from fabrication of a radiographic template to the placement with surgical guide (Jung et al. 2009; Schneider et al. 2009). However, the rigidity of the surgical guide itself and the mobility during placement should be focused as main causes for inaccuracy in this study, because other factors for inaccuracy were almost the same among models prepared for investigations. An acrylic plate fixed on surgical guide gives an additional rigidity to the guide used in the surgery. Moreover, the guide is supported by remaining 11 teeth, 37–44 regions. Therefore, the rigidity should be enough to use the surgical guide for implant surgery in clinical situation. Even though such a condition could be established, the distal part of the surgical guide would be easy to apically sink down, because an anchor of the guide could not be set on distal side of the 45–47 regions for placement in the model. This hypothesis is supported by the results of this study that larger displacements of implants were significantly found in the 47 region than in the 45 region at both the base and the tip. In addition, in regard to the mobility, the whole of the guide would be rolling during placements. Because the remaining teeth are on the 37–44 regions, especially the 45–47, part of the surgical guide would move in mesial, apical, and lingual directions around an axis created by the straight line between the 37 and the 44 during placement. This hypothesis is supported by the results of this study that mean values of displacement were “minus” for the distal and vestibular directions and “plus” for the apical direction at the base and the tip in the 45 and 47 regions. Thus, an anchor of surgical guide should be definitely set on distal side of the 45–47 regions to improve the stability in unilateral free‐end edentulism.

## Conclusion

Within the limitations of this in vitro study, the data for the accuracy of implant surgery with surgical guide would be useful for further studies, because in vitro studies should be substantially made in order to avoid any unnecessary burden of patients, in advance of retrospective or prospective studies. A comparison of the accuracy in this in vitro model between inexperienced and well‐experienced operators should be necessary for clinicians who would intend to use surgical guide for implant placement.

During implant placements, larger displacement of implant position in the 47 region than those in the 45 region suggest that surgical guide might apically sink down. In addition, “minus” values of displacement for the distal and the vestibular directions and “plus” for the apical direction in the 45 and 47 regions suggest that the whole of the guide would be rolling. As the movement might lead to nerve injury in mandible, a strategy for setting anchor of surgical guide on distal side of placement region should be necessary to improve the stability in unilateral free‐end edentulism.

## Conflict of Interest

No conflict of interest.
